# Postnatal Constant Light Compensates *Cryptochrome1* and *2* Double Deficiency for Disruption of Circadian Behavioral Rhythms in Mice under Constant Dark

**DOI:** 10.1371/journal.pone.0080615

**Published:** 2013-11-20

**Authors:** Daisuke Ono, Sato Honma, Ken-ichi Honma

**Affiliations:** 1 Photonic Bioimaging Section, Research Center for Cooperative Projects, Hokkaido University Graduate School of Medicine, Sapporo, Hokkaido, Japan; 2 Department of Chronomedicine, Hokkaido University Graduate School of Medicine, Sapporo, Hokkaido, Japan; University of Lübeck, Germany

## Abstract

Clock genes *Cryptochrome* (*Cry1*) and *Cry2* are essential for expression of circadian rhythms in mice under constant darkness (DD). However, circadian rhythms in clock gene *Per1* expression or clock protein PER2 are detected in the cultured suprachiasmatic nucleus (SCN) of neonatal *Cry1* and *Cry2* double deficient (*Cry1* ^-/-^/*Cry2* ^-/-^) mice. A lack of circadian rhythms in adult *Cry1* ^-/-^/*Cry2* ^-/-^ mice is most likely due to developmentally disorganized cellular coupling of oscillating neurons in the SCN. On the other hand, neonatal rats exposed to constant light (LL) developed a tenable circadian system under prolonged LL which was known to fragment circadian behavioral rhythms. In the present study, *Cry1* ^-/-^/*Cry2* ^-/-^ mice were raised under LL from postnatal day 1 for 7 weeks and subsequently exposed to DD for 3 weeks. Spontaneous movement was monitored continuously after weaning and PER2::LUC was measured in the cultured SCN obtained from mice under prolonged DD. Surprisingly, Chi square periodogram analysis revealed significant circadian rhythms of spontaneous movement in the LL-raised *Cry1* ^-/-^/*Cry2* ^-/-^ mice, but failed to detect the rhythms in *Cry1* ^-/-^/*Cry2* ^-/-^ mice raised under light-dark cycles (LD). By contrast, prolonged LL in adulthood did not rescue the circadian behavioral rhythms in the LD raised *Cry1* ^-/-^/*Cry2* ^-/-^ mice. Visual inspection disclosed two distinct activity components with different periods in behavioral rhythms of the LL-raised *Cry1^-/-^/Cry2^-/-^* mice under DD: one was shorter and the other was longer than 24 hours. The two components repeatedly merged and separated. The patterns resembled the split behavioral rhythms of wild type mice under prolonged LL. In addition, circadian rhythms in PER2::LUC were detected in some of the LL-raised *Cry1^-/-^/Cry2^-/-^* mice under DD. These results indicate that neonatal exposure to LL compensates the CRY double deficiency for the disruption of circadian behavioral rhythms under DD in adulthood.

## Introduction

Circadian rhythms in physiology and behavior in mammals are regulated by coordination of a central clock located in the hypothalamic suprachiasmatic nucleus (SCN) with peripheral clocks in a variety of tissues [1]. The molecular clockwork in the SCN is believed to be conducted by an auto-regulatory transcription and translation feedback loop [2], in which clock genes *Cryptochrome* (*Cry1*) and *Cry2* and their protein products play crucial roles. The latter notion is based on the observation that *Cry1* and *Cry2* double deficient (*Cry1^-/-^/Cry2^-/-^*) mice become aperiodic in behavior immediately after exposure to constant darkness (DD) [3]. However, we [4] and another group [5] reported that significant circadian rhythms of clock gene *Per1* expression and of clock gene product PER2 were detected in the cultured SCN slice of neonatal *Cry1^-/-^/Cry2^-/-^* mice at the cell as well as the tissue levels. The circadian rhythms were also detected in neuronal activity of cultured SCN from these mice [4]. The circadian rhythms at the tissue level disappeared after weaning but those at the cell level persisted until adulthood, indicating that the cellular coupling of circadian oscillation was lost during the postnatal development in *Cry1^-/-^/Cry2^-/-^* mice. Furthermore, circadian PER2 rhythms were restored in the SCN of adult *Cry1^-/-^/Cry2^-/-^* mice by co-culture of the neonatal SCN. The results indicated that the SCN of adult *Cry1^-/-^/Cry2^-/-^* mice still possesses the capability to oscillate in response to a diffusible factor(s) secreted from the neonatal SCN, and that CRY1 and CRY2 are involved in the developmental reorganization of SCN circadian system to sustain cellular couplings of oscillating cells [4].

Prolonged constant light (LL) was known to fragment the circadian behavioral rhythms in rats [6]. However, rats raised under LL in the postnatal period developed the tenable circadian system under prolonged LL in adulthood [7]. LL was also demonstrated to restore the disrupted circadian behavioral rhythms of *Clock* mutant [8] and *Per2/Cry1* double mutant mice [9]. These observations suggest that exposure to LL during the early developmental stage could enhance the coupling strength of the circadian system in the SCN.

In the present study, we examined the effects of prolonged exposure to LL during the early developmental period on the 24 h profiles of spontaneous movement and PER2::LUC in the cultured SCN of adult *Cry1^-/-^/Cry2^-/-^* mice under DD. Surprisingly, the circadian rhythms in behavior were detected in most of the LL-raised *Cry1^-/-^/Cry2^-/-^* mice. In addition, circadian PER2::LUC rhythms were detected in some mice. Visual inspection revealed two activity components of different periods in the LL-raised *Cry1^-/-^/Cry2^-/-^* mice under DD. LL exposure in adulthood failed to rescue the behavioral rhythms in *Cry1^-/-^/Cry2^-/-^* mice. These results indicate that postnatal exposure to LL compensates the CRY double deficiency for the disruption of circadian behavioral rhythms under DD.

## Materials and Methods

### Animals

*Cry1^-/-^/Cry2^-/-^* mice of C57BL/6J background [10] were used for behavior experiments. To measure PER2 in the cultured SCN, *Cry1^-/-^/Cry2^-/-^* mice carrying a PER2 bioluminescence reporter (*Cry1^-/-^/Cry2^-/-^*–LUC) were developed by breeding *Cry1^-/-^/Cry2^-/-^* mice with the PER2::LUC mice carrying a PER2 fusion luciferase reporter [11]. For the control, the wild type (WT) mice carrying a PER2 bioluminescence reporter (WT-LUC) were used [4]. Mice were reared in our animal quarters where environmental conditions were controlled (lights-on 06:00-18:00 h; light intensity approximately 100 lx at the bottom of cage, humidity 60±10%) unless otherwise stated. The day of birth was designated as postnatal day 0 (P0). They were weaned at P21, unless otherwise stated. Animals had free access to commercial chow and tap water.

### Measurement of behavior activities

**Figure 1:**
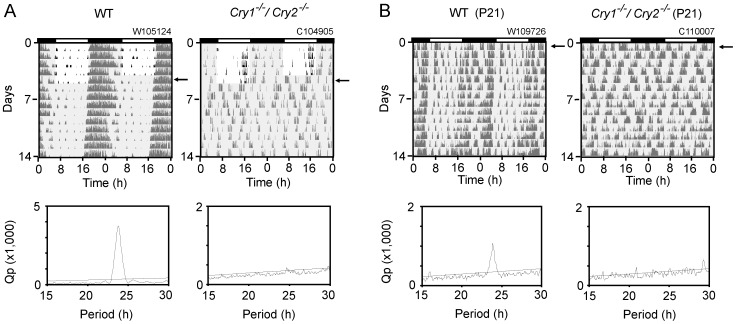
Behavior activities in *Cry1^-/-^/Cry2^-/-^* mice. Representative records of wheel-running activity (A) in an adult WT (left) and *Cry1^-/-^/Cry2^-/-^* (right) mice, and of spontaneous movements (B) in a weanling WT (left) and *Cry1^-/-^/Cry2^-/-^* (right) mice are demonstrated as double-plotted actograms. The ordinate indicates the day of measurement. The gray shaded area in each panel indicates the time of darkness, and arrows indicate the first day of DD. A black and white bar at the top of panel indicates the LD condition (black; dark, white; light) before exposure to DD (A) or before weaning (B). A number on the top of each panel indicates the identification number of an animal. The number at the ordinate indicates the day of recording. The respective Chi square periodograms were obtained using the last 14 day records. An oblique line in a Chi square periodogram indicates a significance level of p = 0.01.

Behavior activities were monitored mostly by a thermal sensor but by a running-wheel in one experiment (Fig.1A). Mice were individually housed in a polycarbonate cage placed in a light-proof box in which ambient temperature was controlled by air-conditioning and a light-dark cycle of LD 12:12 were given with a fluorescence tube of ca. 300 lx in the light phase.

Spontaneous movement was monitored by a thermal sensor equipped on the ceiling of an individual box. The thermal sensor detected a change in the amount of infrared radiation from an animal by movement. Wheel-running activity was measured in a cage with a running-wheel (10 cm in diameter). Spontaneous movements and wheel revolutions were fed into computer every 1 min by computer software (The Chronobiology Kit; Stanford Software System).

Circadian periodicity was determined by Chi square periodogram analysis with the significance level of p = 0.01 (Clock Lab software, Actimetrics) using activity records of 5 min bins. The largest peak with clear rising and falling limbs was taken as the primary period. When the activity band was split into more than one activity component, circadian periods of behavioral components were also determined by an eye-fitted regression line, In the latter case, three experimenters (DO, SH, KH) drew a regression line fitted to prominent activity bouts and calculated a circadian period. When more than one experimenter obtained circadian periods, the mean value was used for further analyses as an individual period.

### SCN slice culture and bioluminescence measurement

A coronal SCN slice of 300 µm thick was made by a microslicer (D.S.K: DTK-1000; Dosaka EM). The tissue containing the middle portion of the SCN was dissected and a paired SCN was cultured on a Millicell-CM culture insert (Millipore Corporations) in a 35-mm Petri dish. The culture was performed in air at 36.5°C with 1.3 ml Dulbecco's modified Eagle's medium (Invitrogen) supplemented with 10 mM HEPES, 2.7 mM NaHCO_3_, 20 mg/L kanamycin (Gibco), 100 µg/mL apo-transferrin (Sigma), 5 µg/mL insulin (Sigma), 100 µM putrescine (Sigma), 20 nM progesterone (Sigma), 30 nM sodium selenite (Gibco) and 0.1 mM D-luciferin K salt (DOJINDO).

Bioluminescence from each cultured tissue was measured for 1 min at 10-min intervals with a photomultiplier tube (Lumicycle; Actimetrics). Bioluminescence intensity was expressed as relative light unit (RLU; counts/min).

### Experimental protocols

#### Behaviors of *Cry1^-/-^/Cry2^-/-^* mice raised under LD and release into DD

Adult *Cry1^-/-^/Cry2^-/-^* (n = 10) and WT mice (n = 6) were reared under LD and subjected to measurement of wheel-running activity at about 8 months old. They were housed individually in a cage with a running-wheel at 2 weeks before released into DD for 3 weeks. In another experiment, *Cry1^-/-^/Cry2^-/-^* (n = 5) and WT (n = 4) mice were raised under LD and weaned at P21. On the day of weaning, they were housed in individual cages and released into DD for 3 weeks. Spontaneous movement was monitored by a thermal sensor from the day of weaning.

#### Spontaneous movement and PER2::LUC in the SCN of *Cry1^-/-^/Cry2^-/-^* mice raised under LL and release into DD

*Cry1^-/-^/Cry2^-/-^* (n = 9) and WT (n = 8) mice were raised under LL from P1 and weaned at P21 or P28, depending on their body weights. On the day of weaning, they were housed in individual cages and continuously exposed to LL for 24 days more. Then they were released into DD for 3 weeks. Spontaneous movement was monitored from the day of weaning.

*Cry1^-/-^/Cry2^-/-^* -LUC (n = 9) and WT-LUC (n = 6) mice were raised under LL from P1 and weaned at P21 or P28 depending on their body weight. On the day of weaning, they were housed in individual cages and continuously exposed to LL for 24 days more. Then they were released into DD for 3 – 4 weeks. At the end of DD, the brains were sampled for culture. Spontaneous movement was monitored from the day of weaning.

#### Spontaneous movement and PER2::LUC in the SCN of LD-raised *Cry1^-/-^/Cry2^-/-^* mice exposed to LL and subsequent DD in adulthood

*Cry1^-/-^/Cry2^-/-^* -LUC (n = 8) and WT-LUC (n = 8) mice were raised under LD until adulthood. At 3 to 4 months old, they were housed individually and exposed to LL for 7 weeks, which was followed by release into DD for 3 – 4 weeks. Spontaneous movement was measured during exposure to LL and DD, and brain was sampled for culture at the end of DD exposure.

### Data analysis and statistics

The raw bioluminescence data were smoothed using a 5 point moving average method. These data were detrended by a 24 h running average subtraction method. The significance of a circadian rhythm was evaluated by Chi square periodogram analysis (ClockLab) with a significance level of P = 0.01. The group mean is presented as the mean ± SD. t-test was used when two independent group means were compared (Statview).

### Ethics statement

Animals were cared for according to the Guidelines for the Care and Use of Laboratory Animals in Hokkaido University. All experiments were approved by Animal Research Committee of Hokkaido University under permission number 08–0279. Animals were euthanized by cervical dislocation, and all efforts were made to minimize suffering.

## Results

### Circadian behavioral rhythms are disrupted under DD in weanling and adult *Cry1^-/-^/Cry2^-/-^* mice

Chi square periodogram analysis confirmed circadian arrhythmicity in wheel-running activity under DD in adult *Cry1^-/-^/Cry2^-/-^* mice which were raised under light-dark cycles (LD) until adulthood. In all *Cry1^-/-^/Cry2^-/-^* mice (n = 10), the behavioral rhythms became arrhythmic immediately after transfer from LD to DD (Fig. 1A). By contrast, 24 h rhythms were still observed under LD, where the activity onset of behavioral rhythm showed a positive phase angle to the light–off time (0.8±1.3 h, n = 10). Similarly, spontaneous movement measured by infrared thermal sensor in the LD-raised *Cry1^-/-^/Cry2^-/-^* mice had no circadian rhythmicity when the mice were released to DD at weaning of postnatal day 21 (P21). Whereas, the behavioral rhythms in the wild-type (WT) mice were observed from the day of weaning (Fig. 1B). The circadian rhythms free-ran with a period slightly shorter than 24 h (23.8±0.1 h, n = 4) (Fig. 1). The results indicate that spontaneous movement was already arrhythmic at weaning in the LD-raised *Cry1^-/-^/Cry2^-/-^* mice.

### Circadian rhythms in *Cry1^-/-^/Cry2^-/-^* mice raised under LL from birth and exposed to DD

#### Spontaneous movement

**Figure 2:**
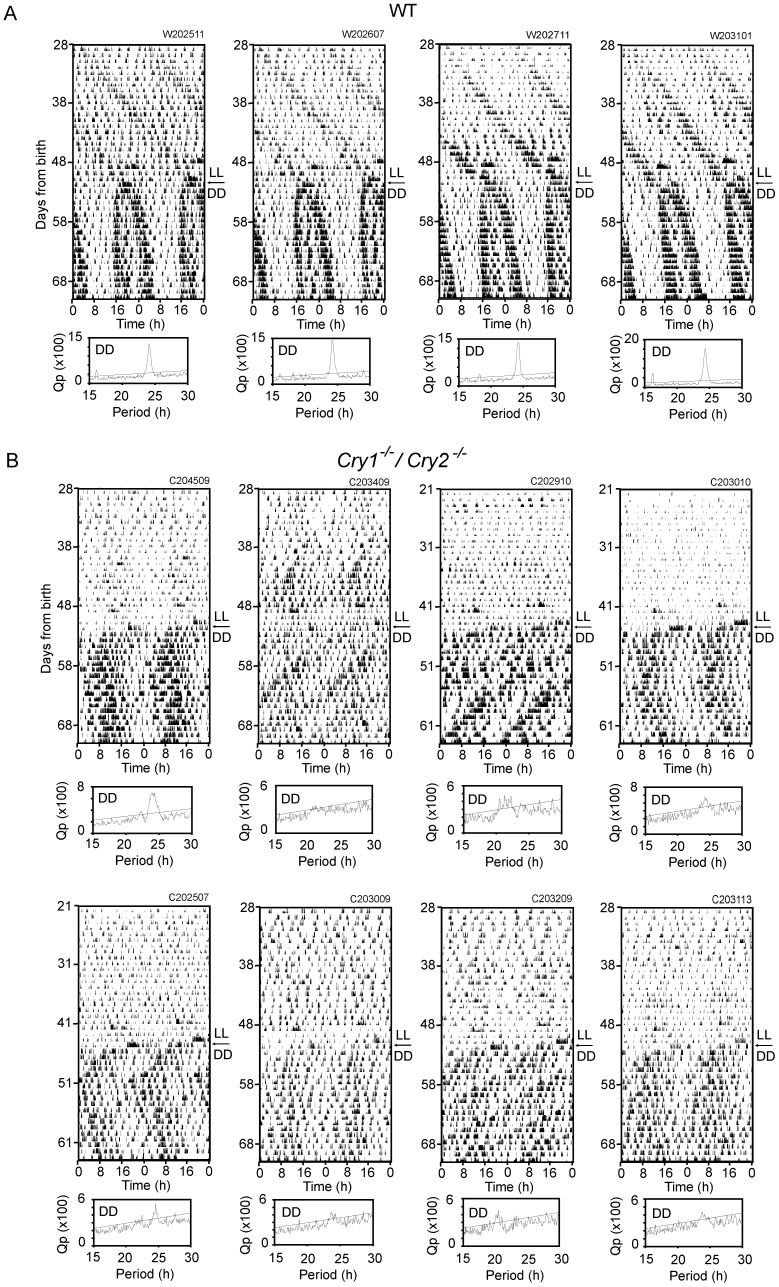
Circadian behavioral rhythms in LL-raised mice under LL and subsequent DD. Circadian rhythms in spontaneous movements are illustrated as a double-plotted actograms for representative four LL-raised WT (A) and eight LL-raised *Cry1^-/-^/Cry2^-/-^* mice (B) which showed two activity components. Arrows indicate the first day of DD. The ordinate indicates the days from birth. Chi-square periodograms for respective records under DD are illustrated. An oblique line in the periodogram indicates a significance level of p = 0.01.

*Cry1^-/-^/Cry2^-/-^* mice (n = 9) were exposed to LL from the postnatal day 1 (P1) for about 7 weeks, and subsequently to DD for 3 weeks. They were weaned at P21. Some mice were weaned at the postnatal day 28 (P28), because of delay in physical development which was estimated by body weight. Mice with low body weight were also found in the LD raised group and they were weaned at P28. Spontaneous movement was measured continuously from weaning and evaluated for circadian periodicities by Chi square periodogram analysis and visual inspection (Table 1). Circadian rhythms were evident in one *Cry1^-/-^/Cry2^-/-^* mouse under LL by Chi square periodogram analysis, and a free-running activity component was detected by visual inspection in some mice (n = 5). On the other hand, significant circadian rhythms were detected in all *Cry1^-/-^/Cry2^-/-^* mice examined under DD (Fig. 2B). The circadian rhythms, however, were not stable in terms of circadian period. They showed sometimes abrupt changes in periodicity. The mean circadian period determined by Chi square periodogram analysis was 23.2±1.8 h in *Cry1^-/-^/Cry2^-/-^* mice. It was not different from that of WT mice (24.2±0.1, n = 8) because of a large SD in *Cry1^-/-^/Cry2^-/-^* mice. By visual inspection, two activity components were identified in spontaneous movement of most *Cry1^-/-^/Cry2^-/-^* mice (n = 8). They free-ran with different circadian periods; a shorter and longer period than 24 h respectively. An eye-fitted regression line revealed that the mean shorter period was 22.8±0.4 h (n = 8) and the mean longer period was 24.6±0.2 h (n = 9). The difference of two periods was statistically significant (p<0.01). Two significant periodicities were also detected by Chi square periodogram analysis in some *Cry1^-/-^/Cry2^-/-^* mice (n = 4). By contrast, circadian rhythms were detected in most WT mice under LL (n = 6) and all mice under DD (n = 8) by Chi square periodogram analysis. The mean circadian periods were 25.6±0.4 h under LL and 24.2±0.1 h under DD. The difference of two periods was statistically significant (p<0.01). On the other hand, visual inspection revealed the circadian rhythms in all WT mice examined under both LL and DD. The mean circadian period was 25.5±0.2 h under LL and 24.1±0.1 h under DD, respectively. The periods determined by visual inspection were not significantly different from the respective values determined by Chi square periodogram analysis. In the wild type mice, the activity band of circadian behavioral rhythm was lengthened (decompressed) upon the transfer to DD, with the activity onset phase-advancing and the end of activity phase-delaying until reaching a steady state phase-relation between them (Fig. 2A).

**Table 1:** Mean circadian periods of spontaneous movement in the LD- and LL-raised *Cry1^-/-^/Cry2^-/-^* mice under LL or DD.

	LL raised mice		LD raised mice	
	WT	*Cry1^-/-^/Cry2^-/-^*	WT	*Cry1^-/-^/Cry2^-/-^*
**LL**	25.6±0.4 (n = 6/8)	23.3 (n = 1/9)	S: 23.5±0.3 (n = 8/8)	-
	(25.5±0.2) (n = 8/8)	(23.7±1.0) (n = 5/9)	S: (23.4±0.3) (n = 8/8)	-
			L: 25.4±0.2 (n = 8/8)	-
			L: (25.4±0.1) (n = 8/8)	-
**DD**	24.2±0.1 (n = 8/8)	23.2±1.8 (n = 9/9)	24.2±0.2 (n = 8/8)	-
	(24.1±0.1) (n = 8/8)	S: (22.8±0.4) (n = 8/9)	(24.1±0.1) (n = 8/8)	-
		L: (24.6±0.2) (n = 9/9)		

Mean circadian periods by Chi square periodogram analysis are listed with SD and those determined by visual inspection are indicated in parentheses. n; the number of significant or detected rhythms per examined. S and L mean short and long period, respectively. -: no significant rhythm was detected.

#### PER2::LUC in the cultured SCN

**Figure 3:**
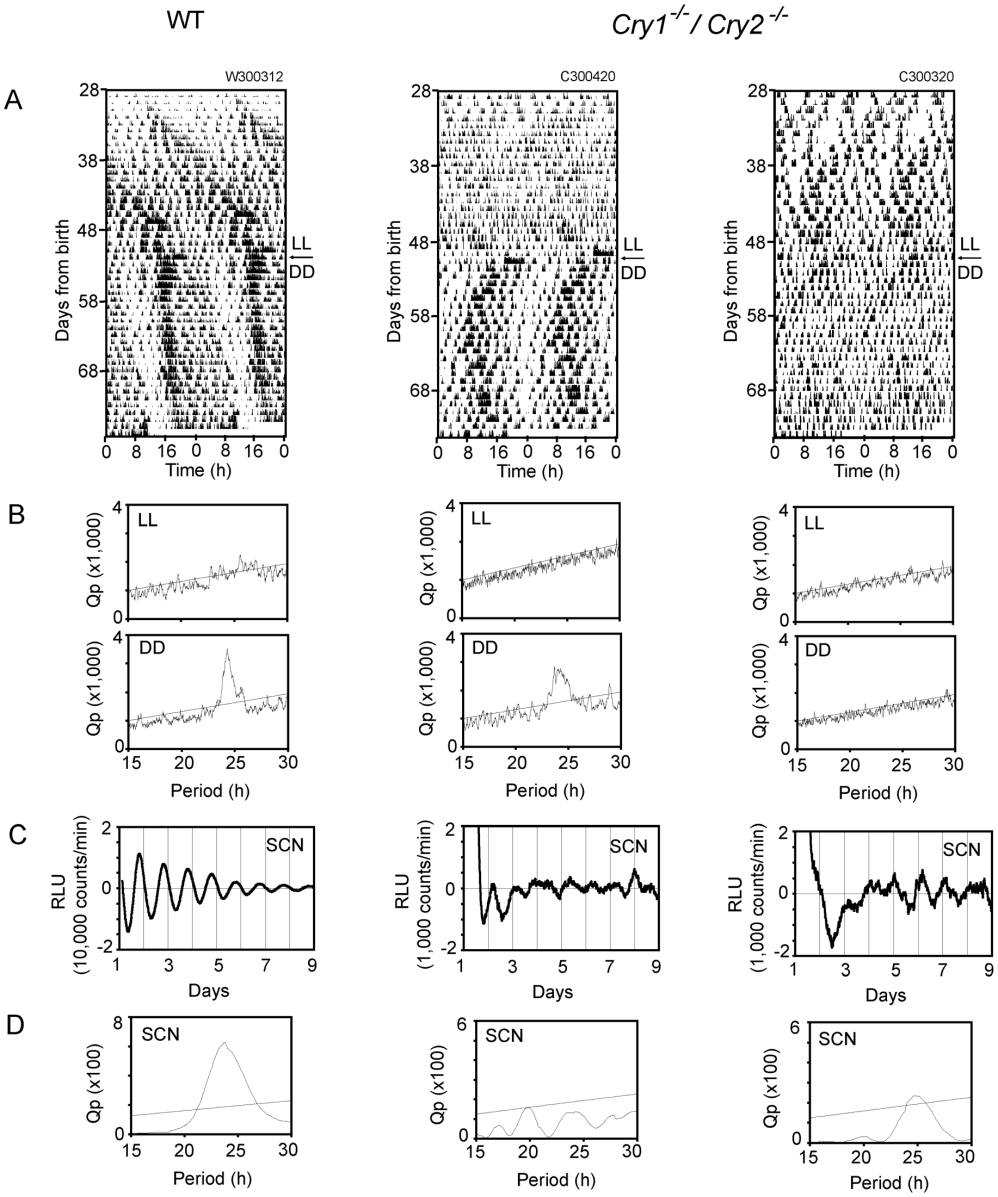
PER2::LUC rhythms in the SCN of LL-raised and DD-exposed mice. Representative circadian rhythms of spontaneous movement (A) and PER2::LUC in the SCN (C) of WT-LUC and *Cry1^-/-^/Cry2^-/-^* -LUC mice are demonstrated. The behavioral rhythm was illustrated as double-plotted actograms (A) with Chi square periodogram (B) under LL (upper) and DD (lower) for WT-LUC and *Cry1^-/-^/Cry2^-/-^* -LUC mice. Respective periodogram for circadian PER2::LUC rhythms was also demonstrated (D). An oblique line in the periodogram indicates a significance level of p = 0.01.

The WT (n = 6) and *Cry1^-/-^/Cry2^-/-^* mice (n = 9) carrying a PER2::LUC reporter system were raised under LL from P1 and transferred to DD 3-4 weeks after weaning with exactly the same protocol as for the above mentioned behavior experiment. Spontaneous movement was also monitored in these mice after weaning (Fig. 3A). The SCN was sampled 3 - 4 weeks after transfer to DD. The SCN slice was cultured on a membrane and the intensity of bioluminescence was measured by a photomultiplier tube (Fig. 3C).

Circadian rhythms were evaluated by Chi square periodogram analysis. Significant circadian rhythms of PER2::LUC were observed in all SCN from WT mice. By contrast, circadian PER2::LUC rhythms were detected only in 3 out of 9 SCN from *Cry1^-/-^/Cry2^-/-^* mice (Fig. 3D). The circadian PER2::LUC rhythms in *Cry1^-/-^/Cry2^-/-^* mice showed low amplitudes and sometimes two peaks within a circadian cycle (bimodality).

In these mice, significant circadian rhythms in spontaneous movement were detected by Chi square periodogram analysis in all WT mice examined but only in 2 out of 9 *Cry1^-/-^/Cry2^-/-^* mice (Fig. 3B). Visual inspection, however, indicated two activity components in most *Cry1^-/-^/Cry2^-/-^* mice (n = 7) with a shorter and longer period than 24 h. The mean circadian periods were 22.8±0.4 h (n = 7) and 24.7±0.3 h (n = 7), respectively. The two circadian periods were significantly different (p<0.01).

Inconsistency was detected in the PER2::LUC rhythm. Significant circadian rhythms were detected in some mice, whereas bimodality and even arrhythmicity were observed in others. Discrepancy was also noticed in the circadian rhythm expression between behavior and PER2::LUC in some individuals, as illustrated in Fig. 3. The circadian rhythm in spontaneous movement was detected by Chi square periodogram immediately before the harvest of the SCN (Fig. 3A and B, middle), while the circadian PER2::LUC rhythm was not detected in the subsequent culture of the SCN (Fig. 3C and D, middle). On the other hand, in some mice the circadian rhythm in *Per2* expression was detected in the cultured SCN (Fig. 3C and D, right), while behavior was arrhythmic immediately before harvest (Fig. 3A and B, right).

### Circadian behavioral rhythms were not restored in *Cry1^-/-^/Cry2^-/-^* mice exposed to LL in adulthood

**Figure 4:**
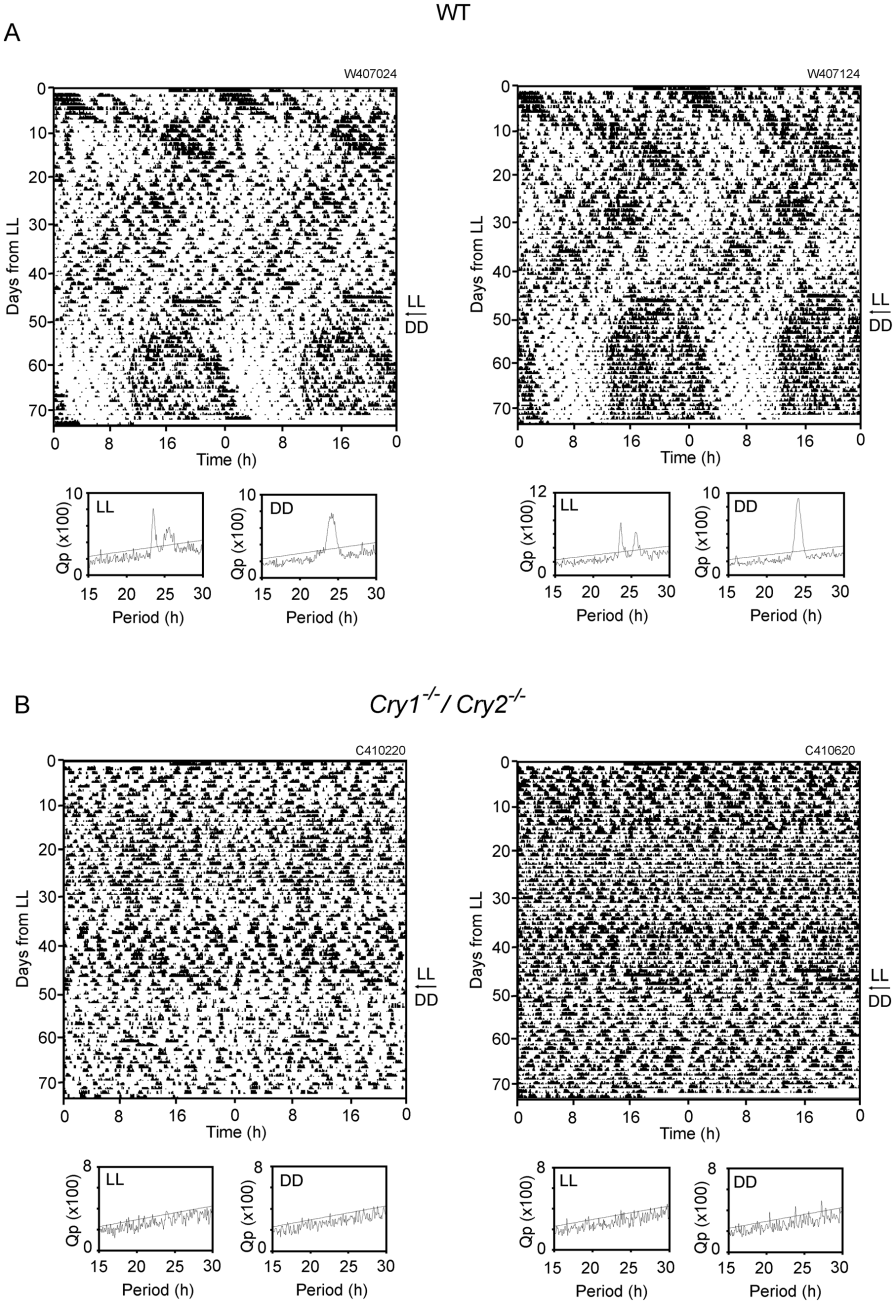
Circadian behavioral rhythms in the LD-raised mice under LL and subsequent DD in adulthood. Circadian behavioral rhythms in LD-raised WT-LUC (A) and *Cry1^-/-^/Cry2^-/-^* -LUC mice (B) under LL and DD are demonstrated as double-plotted actograms. Respective Chi square periodograms are illustrated for the rhythms under LL (left) and DD (right). An oblique line in the periodogram indicates a significance level of p = 0.01.

Adult *Cry1^-/-^/Cry2^-/-^* and WT mice raised under LD were exposed to LL for 7 weeks and subsequently to DD for 3 - 4 weeks. By Chi square periodogram analysis, a circadian rhythm in spontaneous movement was not detected in *Cry1^-/-^/Cry2^-/-^* mice either under LL or under subsequent DD (Fig. 4B, Table 1). On the other hand, two activity components of different periods were detected in WT mice under LL (Fig. 4A). One period was shorter than 24 h and the other was longer than 24 h. The two activity components were crossing at about 10 day intervals. At the time of crossing, the level of activity seemed to increase. Two activity components were detected in all WT mice examined (n = 8) by both Chi square periodogram analysis and visual inspection (Table 1). The mean circadian period of an activity component with a shorter period was 23.5±0.3 h by Chi square periodogram analysis and 23.4±0.3 h by visual inspection, and those of an activity component with a longer period was 25.4±0.2 h by Chi square periodogram analysis and 25.4±0.1 h by visual inspection, respectively. The short and long circadian periods were significantly different (p<0.01), regardless of analytical methods. Furthermore, the long and short circadian periods of WT mice were significantly longer than the respective periods of the LL-raised *Cry1^-/-^/Cry2^-/-^* mice under DD (p<0.01). The circadian period of the adult WT mice under DD was 24.2±0.2 h by Chi square periodogram analysis and 24.1±0.1 h by visual inspection. And those of the LL-raised WT mice under DD were 24.2±0.1 h by Chi square periodogram analysis and 24.1±0.1 h by visual inspection. The four circadian periods were not significantly different.

## Discussion

Circadian rhythms in spontaneous movement of most *Cry1^-/-^/Cry2^-/-^* mice were detected under DD when exposed to LL from P1. Circadian rhythms were also detected in PER2::LUC in the cultured SCN of some LL-raised *Cry1^-/-^/Cry2^-/-^* mice. Visual inspection revealed two activity components of different periods in behavior. By contrast, the circadian rhythms failed to develop in *Cry1^-/-^/Cry2^-/-^* mice when raised under LD and exposed to LL in adulthood. These findings indicate that neonatal exposure to LL compensates the CRY double deficiency for the disruption of circadian rhythms under DD.

The present results challenge the central idea of molecular circadian clocks in mice that the clock genes, *Cry1* and *Cry2*, are essential components of the auto-regulatory feedback loop for circadian rhythm generation. The notion is primarily based on the finding that the circadian rhythms were lost in *Cry1^-/-^/Cry2^-/-^* mice under DD [3], [12]. The present results are consistent with the recent findings that significant circadian rhythms in clock gene expression were detected in cultured SCN slices obtained from neonatal *Cry1^-/-^/Cry2^-/-^* mice [4], [5], and that the circadian rhythms in the cultured SCN were restored in adult *Cry1^-/-^/Cry2^-/-^* mice by co-culture of the SCN slice from WT mice [4].

Two distinct behavioral components were observed in the LL-raised *Cry1^-/-^/Cry2^-/-^* mice under DD but not always evident. Either of the components sometimes became vague. In addition, the behavior activity was enhanced when two components crossed. These complex expressions of behavior produced some inconsistency in the determination of circadian period between Chi square periodogram analysis and eye-fitted regression line. Nevertheless, the pattern was quite similar to the behavioral rhythms in WT mice exposed to prolonged LL in adulthood. In the latter case, two activity components were detected clearly by Chi square periodogram analysis as well. The circadian periods of each component were significantly longer in WT than in *Cry1^-/-^/Cry2^-/-^* mice by ca. 2 h. The difference could be attributable to the background illumination (DD vs LL).

The changes of circadian behavioral rhythm induced by prolonged exposure of nocturnal rodents to LL are well known: lengthening the circadian period, splitting of the activity band into two components as in the LD-raised WT mice, and abolishment of circadian rhythm [6], [7]. These changes were explained by uncoupling of constituent oscillators in the circadian system. Based on splitting of behavioral rhythm into two activity components, Pittendrigh [13] advanced the hypothesis that the circadian system consists of two oscillators referred as the evening (E) and morning (M) oscillators which drive an evening component (onset of activity) and morning component (end of activity) of circadian behavioral rhythm. Two distinct activity components observed in the LL-raised *Cry1^-/-^/Cry2^-/-^* mice may correspond to the morning and evening components of circadian behavioral rhythm.

Increase of the light sensitivity was suggested in *Cry1^-/-^/Cry2^-/-^* mice, since light exposure completely suppressed activity bouts that were expressed under DD [3]. Previously, Canal-Corretger et al [7] reported that neonatal rats developed a tenable circadian system under prolonged LL in adulthood, when they were raised under LL. The robustness was not obtained when exposed to LL in adulthood. These findings are consistent with the present results in the LL-raised *Cry1^-/-^/Cry2^-/-^* mice. Development of a robust circadian system could be due to the decreased sensitivity to light, since prolonged LL is known to degenerate the retinal photoreceptor cells [14]. However, this mechanism does not fully explain the present findings, since the circadian rhythms were not rescued by exposure to LL in adulthood, by which the retinal degeneration is similarly expected [15]. The postnatal exposure to LL was reported to decrease the basal levels of AVP and VIP immunostaining in the SCN, suggesting direct effects on the SCN circadian system [16]. The reorganization of the circadian system in the SCN is likely involved in the development of circadian behavioral rhythm in the LL-raised *Cry1^-/-^/Cry2^-/-^* mice.

Circadian PER2::LUC rhythms were observed in some, but not all LL-raised *Cry1^-/-^/Cry2^-/-^* mice. The inconsistency is likely due to SCN regional circadian rhythms which uncoupled under particular conditions. Previously, we demonstrated two distinct regional rhythms in the cultured SCN [17]. They corresponded most likely to the E and M oscillators respectively. The regional circadian rhythms consisted of multiple cellular rhythms, and their phase-angle difference was changed according to photoperiod in parallel with compression and decompression of activity rhythm. Therefore, it is surmised that the circadian PER2::LUC rhythm in the SCN reflects those of two regional rhythms and could be modified depending on their phase-relationship. As a result, the PER2::LUC in the SCN of *Cry1^-/-^/Cry2^-/-^* mice showed various patterns of periodicities.

In some animals, a discrepancy in circadian rhythm development was detected between behavior and PER2::LUC (Fig. 3). This could be due to different involvement of CRY(s) in these rhythms. However, the discrepancy was also likely due to alternating split and merge of behavioral components and SCN regional oscillators. Since the behavioral rhythm was evaluated throughout the DD period while the PER2::LUC rhythm was at a particular short period, the time difference of rhythm evaluation could produce the discrepancy.

In conclusion, prolonged exposure to LL during the postnatal period restored circadian rhythm in PER2::LUC as well as spontaneous movement in *Cry1^-/-^/Cry2^-/-^* mice under DD. The restored circadian rhythms showed two activity components with different circadian periods, which resembled the split circadian rhythm under prolonged LL in adulthood. The postnatal LL compensates CRY double deficiency for disruption of the circadian system in the SCN by reorganizing the system.
